# Miscellany

**Published:** 1888-12

**Authors:** 


### Miscellany.

### A Proposed Bill for a Patho-Biological Laboratory, to be Presented to Congress for the Encouragement of Scientific Research.

A bill for the establishment of a national patho-biological laboratory, for study and investigation into the nature and causes of contagious and infectious diseases which threaten and endanger the health of the people and the live stock interests of the country.

Be it enacted by the Senate and the House of Representatives of the United States of America, in Congress assembled:

That for the purpose of the better protection to the health of the people of the United States from the ravages of contagious, infectious and malarial diseases, and for the preservation and protection of the great live stock interests of the country from the decimating devastations of pestiferous diseases of a similar nature, and for the more complete elucidation of the relation existing between many diseases of our domestic animals, and the life and health of human beings whose business calls them into intimate relations with them, and of the welfare of the public as consumers of animal food or animal products, there shall be established at Washington, in the District of Columbia, and the United States of America, a laboratory for the purpose of making a continuous and scientific study into the causes and nature of the classes of disease herein mentioned, and of all subjects connected therewith, bearing either upon the public health or animal economies of the country, and that said laboratory be known as the patho-biological laboratory of the United States of America.

It is further enacted, that the general supervision and control of said patho-biological laboratory shall be in the hands of the surgeon-general of the marine hospital and quarantine service of the government of the United States; that the said surgeon-general shall, at the completion of the buildings and grounds herein provided for, appoint two distinct and independent directors, who shall be the heads of two distinct and independent departments of investigation, and who shall be known respectively as the director of the humano-patho-biological, and the zoo-patho-biological institutes of the patho-biological laboratory of the United States. The directors of the institutes named above shall be competent and skilled patho-bacteriologists, the one in human, the other in animal diseases, and shall, respectively, be graduates from a thoroughly organized and world-accredited medical and veterinary school, college, or department of some university, and shall have both been engaged in and have published in some accredited journal or report, investigations which have gained them each reputation in the medico-scientific world, and have demonstrated their fitness for the positions and responsibilities herein provided for.

The salaries of the directors of the institutions above mentioned shall be five thousand dollars ($5,000) per annum.

The said surgeon-general, with and by the advice and consent of the directors of the institutes named, shall also appoint an assistant to each at a salary of twenty-five hundred dollars ($2,500) per annum, respectively; the one assistant to be a creditably graduated doctor ot medicine, and the other a doctor of veterinary medicine, both of whom shall be citizens of the United States and shall have done creditable work in the field of patho-bacteriological investigation.

The directors of the institute named shall appoint, with and by the consent of the surgeon-general of the United States marine hospital service, such other assistants and servants as shall be necessary to carry on the work herein provided for.

The said surgeon-general shall also appoint to each of the institutes named a competent chemist, each of whom shall be fully equal to conducting investigations in search of the true character and value of the ptomaines or toxins produced in the evolution of micro-organismal life, and who shall have previously distinguished themselves in this line of research. The salary of said chemist shall be five thousand dollars ($5,000) each per annum. The surgeon-general aforesaid is also authorized to employ such assistants and servants for the aid of the said chemists as shall be necessary to the faithful and thorough conducting of their investigations.

The salaries of the persons herein named in connection with the work of patho-biological laboratory of the United States shall be paid out of the funds in the treasury upon warrants drawn by the said surgeon-general, countersigned by the secretary of the treasury department of the United States. It is further provided, that in order to stimulate and encourage original research into the cause and nature of the classes of disease herein mentioned, that the said surgeon-general is hereby authorized to offer the free use of such rooms and appurtenances as shall be provided therefor in the said laboratory, to a limited number of such citizens of the United States as may volunteer their services, at their own expense, and who are known to the said surgeon-general to have been creditable graduates of an honorable veterinary or medical school or college, and to have distinguished themselves by original research in the lines of work herein provided for, the necessary room, materials and appliances to be supplied by the officials of said laboratory; said voluntary workers shall agree, in writing, with the said surgeon-general, to accept two young medical or veterinary students of American schools or colleges, to be selected by the said surgeon-general as assistants and students, and to instruct them in the lines of work they may be engaged upon. Such students must be at their own expense, except as to the instruments and implements necessary to work with. The said volunteer investigators must furthermore agree, in writing, to continue any given series of investigation until completed, to the satisfaction of the said surgeon-general, unless otherwise excused thereby, and must further agree to engage upon any special line of research that may be deemed necessary by the said surgeon-general, or the director of the institute, of this laboratory, to which they may have been detailed. They must further agree that all the results and benefits of any work performed by them in any department in this laboratory shall become the property of the government of the United States of America, and be reported in a full and exact manner for publication in the reports of the same. With and by the consent of the director of the institute of this laboratory, in which such a volunteer may be working, and with the written agreement of the surgeon-general aforesaid, such volunteer investigators may have the privilege of publishing advance reports of their work in the medical or scientific journals of this country. It is further provided, that the sum of five hundred and fifty thousand dollars ($550,000) be hereby appropriated from funds in the treasury of the United States, for the purpose of purchasing the necessary land in the City of Washington, and for grading and fencing the same, and for the erection of the necessary buildings thereon and the equipment of the same, in accordance with the uses herein provided for, to-wit:

For the purchase, grading and fencing the land, one hundred and fifty thousand dollars ($150,000). To a laboratory building, two hundred thousand dollars ($200,000).

The necessary apparatus and equipment of the same, one hundred thousand dollars ($100,000). For stables, pens, cages, etc., and residences for grooms and servants, one hundred thousand dollars ($100,000).

It is further provided, that the secretary of the treasury of the United States, the secretary of agriculture, and the surgeon-general of the marine hospital service shall constitute the board of trustees and building and purchasing committee of the patho-biological laboratory of the United States, and that the persons named are hereby empowered to purchase a location, secure the necessary plans, and build and equip the said institution in the manner herein provided for, and to draw the necessary warrants for the payment of the same in accordance with the letter of this act.

---

### *TO MEDICAL MICROSCOPISTS.*

In behalf of "The American Association for the Study and Cure of Inebriety," the sum of $100.00 is offered by Dr. L. D. Mason, Vice-President of the Society, for the best original essay on "The Pathological Lesions of Chronic Alcoholism Capable of Microscopic Demonstration."

The essay is to be accompanied by carefully prepared microscopic slides, which are to demonstrate clearly and satisfactorily the pathological conditions which the essay considers.

Conclusions resulting from experiments on animals will be admissible. Accurate drawings or micro-photographs of the slides are desired.

The essay, microscopic slides, drawings or micro-photographs are to be marked with a private motto or legend and sent to the chairman of the committee on or before October 1, 1890.

The object of the essay will be to demonstrate: *First*, Are there pathological lesions due to chronic alcoholism? *Secondly*, Are these lesions peculiar or not to chronic alcoholism?

The microscopic specimens should be accompanied by an authentic alcoholic history, and other complications, as syphilis, should be excluded.

The successful author will be promptly notified of his success, and asked to read and demonstrate his essay personally or by proxy, at a regular or special meeting of the Medical Microscopical Society of Brooklyn. The essay will then be published in the ensuing number of *The Journal of Inebriety* (T. D. Crothers, Hartford, Conn.,) as the prize essay, and then returned to the author for further publication or such use as he may desire. The following gentlemen have consented to act as a committee:

*Chairman*—W. H. BATES, M.D., F.R.M.S., Lon., Eng.,
(Pres. Med. Microscopical Soc., Brooklyn.)
175 REMSEN ST., BROOKLYN, N. Y.
JOHN E. WEEKS, M. D.,
43 WEST 18TH STREET, NEW YORK.
RICHMOND LENNOX, M. D.,
164 MONTAGUE ST., BROOKLYN, N. Y.

---

EUGENIA, ex-Empress of France, it is reported, has given directions in her will that, at her decease, her body shall be cremated.

